# A Gene-Based Machine Learning Classifier Associated to the Colorectal Adenoma—Carcinoma Sequence

**DOI:** 10.3390/biomedicines9121937

**Published:** 2021-12-17

**Authors:** Antonio Lacalamita, Emanuele Piccinno, Viviana Scalavino, Roberto Bellotti, Gianluigi Giannelli, Grazia Serino

**Affiliations:** 1 National Institute of Gastroenterology “S. de Bellis”, Research Hospital, Castellana Grotte, 70013 Bari, Italy; antonio.lacalamita@irccsdebellis.it (A.L.); emanuele.piccinno@irccsdebellis.it (E.P.); viviana.scalavino@irccsdebellis.it (V.S.); gianluigi.giannelli@irccsdebellis.it (G.G.); 2 Dipartimento Interateneo di Fisica, Università degli Studi di Bari Aldo Moro, 70126 Bari, Italy; roberto.bellotti@ba.infn.it; 3 Istituto Nazionale di Fisica Nucleare, Sezione di Bari, 70125 Bari, Italy

**Keywords:** colorectal cancer, adenoma, machine learning, transcriptomics

## Abstract

Colorectal cancer (CRC) carcinogenesis is generally the result of the sequential mutation and deletion of various genes; this is known as the normal mucosa–adenoma–carcinoma sequence. The aim of this study was to develop a predictor-classifier during the “adenoma-carcinoma” sequence using microarray gene expression profiles of primary CRC, adenoma, and normal colon epithelial tissues. Four gene expression profiles from the Gene Expression Omnibus database, containing 465 samples (105 normal, 155 adenoma, and 205 CRC), were preprocessed to identify differentially expressed genes (DEGs) between adenoma tissue and primary CRC. The feature selection procedure, using the sequential Boruta algorithm and Stepwise Regression, determined 56 highly important genes. K-Means methods showed that, using the selected 56 DEGs, the three groups were clearly separate. The classification was performed with machine learning algorithms such as Linear Model (LM), Random Forest (RF), k-Nearest Neighbors (k-NN), and Artificial Neural Network (ANN). The best classification method in terms of accuracy (88.06 ± 0.70) and AUC (92.04 ± 0.47) was k-NN. To confirm the relevance of the predictive models, we applied the four models on a validation cohort: the k-NN model remained the best model in terms of performance, with 91.11% accuracy. Among the 56 DEGs, we identified 17 genes with an ascending or descending trend through the normal mucosa–adenoma–carcinoma sequence. Moreover, using the survival information of the TCGA database, we selected six DEGs related to patient prognosis (SCARA5, PKIB, CWH43, TEX11, METTL7A, and VEGFA). The six-gene-based classifier described in the current study could be used as a potential biomarker for the early diagnosis of CRC.

## 1. Introduction

Colorectal cancer (CRC) is the third most common malignancy among adults and the second leading cause of cancer-related death worldwide [1]. CRC develops in a linear progression from a normal colonic epithelium to the onset of adenoma, carcinoma transformation, and metastasis [2]. The progression of CRC from adenoma occurs via the accumulation of multiple genetic mutations, epigenetic defects, and other environmental factors over an average time of 10–15 years [3]. Several studies have highlighted that an increased adenoma detection rate has been associated to a reduction in the risk of invasive CRC and mortality [4,5,6]. Thus, the early detection of CRC and precancerous lesions, such as adenomatous polyps, is particularly important. Currently, stool-based screening is the common test used for early CRC detection worldwide. However, these screening tests are unsuitable for adenoma screening due to their poor sensitivity for adenomatous lesions [7]. Colonoscopy, as the gold standard, is not ideal as a screening test because it is invasive and requires bowel preparation and dietary modification, which can cause complications and poor compliance. A highly sensitive screening test for adenoma identification is urgently needed.

Advances in omics technologies have helped to better understand the molecular mechanisms that discriminate normal and tumor tissues at gene, mRNA, and protein levels. Genome-wide gene expression profiling analyses of large numbers of multiple CRC tumor specimens have enabled the classification of four consensus molecular sequences (CMS1–4) with a distinct biology and gene expression patterns, and subtypes characterized by mixed transition features [8]. A comparison between primary lesions and metastatic tumors identified a signature specific to metastatic CRC [9]. However, the classification associated to adenoma is unknown. To date, few studies have been performed to identify gene expression profiling associated to adenoma. As a consequence, the specific expression patterns of these genes during the onset of CRC remain unclear.

The aim of the present study was to develop a predictor-classifier of the stages during the “adenoma-carcinoma” sequence using microarray gene expression profiles of primary CRC, adenoma, and normal colon epithelial tissues. Our study is based on the generation of a decision support system to evaluate the existence of a relationship between the gene expression in the three groups. Our hypothesis is that gene expression could accurately predict not only the presence of the disease, but also its severity (Adenoma or CRC).

## 2. Materials and Methods

### 2.1. Data Description

We analyzed raw microarray data in four different datasets downloaded from the Gene Expression Omnibus database (GEO, https://www.ncbi.nlm.nih.gov/geo, accessed on 24 May 2021) [10]:

- GSE100179: based on GPL17586 Affymetrix Human Transcriptome Array 2.0 platform (Affymetrix; Thermo Fisher Scientific Inc., Waltham, MA, USA). Biopsy samples were collected from 20 healthy colon biopsies (Control), 20 colorectal adenoma (Adenoma) and 20 colorectal cancer (CRC) tissues during routine colonoscopy [11];
- GSE117606: based on GPL25373 Affymetrix HT HG-U133+ PM Array Plate. FFPE samples derived from patients with adenoma, tumor, and adjacent tissues. This set contained 71 CRC, 62 Adenoma, and 65 Control samples [12];
- GSE4183: based on GPL570 Affymetrix Human Genome U133 Plus 2.0 Array. Total RNA was extracted, amplified and biotinylated from frozen colonic biopsies of 15 patients with CRC, 15 with Adenoma, and 8 healthy Controls [13];
- GSE71187: based on GPL6480 Agilent-014850 Whole Human Genome Microarray 4x44K G4112F (Agilent Technologies, Santa Clara, CA). This set contained 99 CRC, 58 Adenoma, and 12 Control samples [14].

In short, the merged dataset consists of 465 samples divided into three cohorts: 105 samples as Healthy Controls; 155 samples in the Adenoma Group; 205 samples in the CRC Group.

### 2.2. Study Design

**Figure 1:**
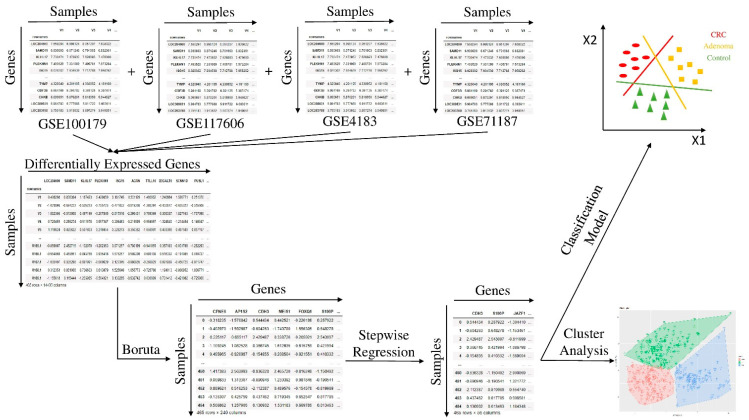
Flowchart of the proposed approach. After merging the four datasets, we implemented a differential expression analysis and a feature selection procedure. Then, we developed a Cluster analysis to evaluate dataset homogeneity, and finally we selected the best learning model to classify the samples into the three groups.

To evaluate a possible relation between the gene expression in the three groups, we proposed an approach based on six main steps, shown in Figure 1:

1. Data preprocessing;
2. Differential Expression Analysis;
3. Feature Selection;
4. Unsupervised learning: Cluster analysis;
5. Supervised learning: Comparison of different classifiers;
6. Test of the most performing learning model.

### 2.3. Data Pre-Processing

To read and preprocess raw data, different packages in the framework R (https://www.r-project.org/, accessed on 24 May 2021) [15] were used. The four datasets were derived from two different technologies: Affymetrix and Agilent. To process Affymetrix CEL files, we used the ‘oligo’ [16] package. The Expression Set was obtained from the CEL files as the result of the Robust Multi-array Average (RMA) algorithm [17], which is a normalization procedure for microarrays that corrects background, normalizes, and summarizes raw intensity values using median-polish. To read and analyze txt files from the Agilent platform, we used the ‘limma’ [18] package. For each spot, the background intensity was subtracted from the foreground intensity using the “background Correct” function and the dataset was normalized by “normalize Between Arrays”. Then, after normalization of the four datasets, we added the gene annotation. The signal intensities of multiple probes that represent a unique gene were used as mean value. Since the datasets derived from different microarray technologies, we considered the signal intensities associated to each gene instead of the probe and each dataset was standardized using z-score normalization. Briefly, the mean intensities of all features were subtracted from the relative intensity value of each gene and the difference was divided by the standard deviation.

### 2.4. Differential Expression Analysis

Differential expression analysis was performed to find genes that are differentially expressed (DEG) in different conditions. To study DEGs, an unpaired *t*-test, included in the ‘limma’ [18] package in R, was used. The following comparisons were carried out: CRC vs. Control, Adenoma vs. Control, and CRC vs. Adenoma. The limma approach starts by fitting a linear model to the preprocessed data and then using an empirical Bayes method to moderate the standard errors of the estimated log-fold changes [19]. In this analysis, a DEG was defined as Log Fold Change: |log2 FC| ≥ 0.263; False Discovery Rate: (FDR) < 0.05.

### 2.5. Feature Selection

Starting from the list of DEGs for all comparisons, we performed a feature selection procedure to select a subset of relevant features for the construction of the model. To select the best DEGs on which to develop a gene-based classifier for the adenoma–CRC evolution, we based the feature selection on the sequential use of two algorithms: firstly Boruta [20] and then Stepwise Regression [21]. The Boruta algorithm, present in the ‘Boruta’ package [20] in R, uses a wrapper approach built around a Random Forest [22] classifier to perform a robust, supervised feature selection. Boruta is based on the same idea that forms the basis of the random forest classifier. Briefly, by adding randomness to the system and collecting results from the ensemble of randomized samples, the misleading impact of random fluctuations and correlations can be reduced [20]. Stepwise Regression is a step by step method that studies the statistical significance (compared to a selected criterion) of each feature through a linear regression model. It is a combination of the forward and backward selection techniques [21]. In each step, a variable is considered for addition to, or subtraction from, a set of explanatory variables, based on an information criterion. We used the ‘caret’ package [23] in R to implement this method. Finding the subset of independent regressor variables involves two opposing objectives. In this study, we used the Bidirectional Elimination procedure, which is a combination of the forward and backward selection techniques.

### 2.6. Unsupervised Learning: Clustering Analysis

Following the feature selection procedure, we studied the dataset homogeneity through an unsupervised learning method. This approach permits associations and patterns among the set of input variables to be identified, verifying whether gene expression is able to characterize the three groups. Here, we used the clustering procedure for the unsupervised learning method. In detail, we performed the clustering procedure in three steps, as follows: (1) Optimal cluster number k evaluation: Silhouette [24], Within Cluster Sum of Squares [25], and Gap Statistic Method [26]; (2) Ward hierarchical cluster [27] development, dendrogram cut in k cluster, and centroids estimation; (3) Sample grouping in k cluster through the K-Means clustering using the centroids, estimated in step 2, as algorithm initialization.

This procedure had been implemented using the ‘factoextra’ [28] package for step 1 and the basic R packages for the other two steps.

### 2.7. Supervised Learning: Classification Model

Simultaneous to the clustering analysis, we implemented Supervised Learning methods, analyzing the training data that produce an inferred function, which can be used for mapping new examples. We compared the performances of four different classifier algorithms: Linear Model (LM), Random Forest (RF), k-Nearest Neighbors (k-NN), and Artificial Neural Network (ANN). They had been fed with the features selected by the Stepwise Regression algorithm. We started with a linear hypothesis and then applied three different machine learning algorithms based on different fundamental units, decision trees for RF, and artificial neuron for ANN. In order to build a robust classifier, we randomly chose 90 observables (about 20% of the entire dataset), 30 for each class, and used them as validation set. The four predictors were built on the remaining 375 observables.

The classification models had been implemented using basic R package for LM, ‘randomForest’ [29] package in R for RF, function ‘knn3′ present in the ‘caret’ [23] package in R for k-NN, and ‘neuralnet’ [30] package in R for ANN.

### 2.8. Cross-Validation and Performance Metrics

The robustness of the classifier was verified by performing a cross-validation technique. Specifically, we applied k-fold cross-validation that randomly partitioned the original sample into k equal-sized subsamples. On the k subsamples, a single subsample was retained as the validation datum for testing the model, and the remaining k-1 subsamples are used as training data. The cross-validation process was then repeated several times, with each of the k subsamples being used once as the validation datum. The four models had been compared by applying a common five-fold cross-validation, repeated 300 times in order to avoid overfitting and to evaluate the model stability. The validation set was used to confirm the transferability and goodness of the classifier.

The performances of the classification model were assessed with the following parameters:

The Area Under the Curve (AUC) is the measure of the ability of a classifier to distinguish between classes and is used as a summary of the ROC curve, estimated by the ‘multiclass.roc’ function from the ‘pROC’ [31] package in R.

Accuracy is the proportion of true results among the total number of cases examined. We defined *TP_i_*, *TN_i_*, *FP_i_*, *FN_i_* as true positives, true negatives, false positives, and false negatives, respectively, in a classification problem with *N* classes, the accuracy for the class *i* is defined as:

(1) $$Acc_{i}=\frac{TP_{i}+TN_{i}}{TP_{i}+TN_{i}+FP_{i}+FN_{i}}$$

The total accuracy is the average of all class values; it was evaluated by the function ‘Accuracy’ from the ‘ML metrics’ [32] package in R.

Sensitivity is the fraction of the total amount of relevant observations that were actually retrieved:

(2) $$Sens_{i}=\frac{TP_{i}}{TP_{i}+FN_{i}}$$

Precision is the fraction of relevant observations among the retrieved observations:

(3) $$Prec_{i}=\frac{TP_{i}}{TP_{i}+FP_{i}}$$

*F*1 score is the harmonic mean of precision and sensitivity:

(4) $$F1_{i}=2\cdot \frac{Prec_{i}\cdot Sens_{i}}{Prec_{i}+Sens_{i}}$$

where all these three metrics were studied through the ‘confusion Matrix’ function from the ‘caret’ [23] package in R.

In order to detect significant differences among the four algorithms, the Kruskal–Wallis test [33] was applied.

### 2.9. Gene Set Enrichment Analysis

Gene Set Enrichment Analysis (GESA) was performed with GSEA software version 4.1.0 (https://www.gsea-msigdb.org/gsea/index.jsp, accessed on 24 May 2021) using the hallmark gene sets of the Molecular Signature Database gene set version 7.4 [34]. Phenotype permutations were performed with a permutation number of 1000. Hallmarks were selected using the FDR value threshold of 0.05. Gene Ontology (GO) analysis and functional pathways analysis were conducted with g:Profiler (https://biit.cs.ut.ee/gprofiler/gost, accessed on 24 May 2021) [35].

## 3. Results

### 3.1. Differential Expression Analysis and Feature Selection

Before using the feature selection algorithms, we performed differential expression analysis to screen the dataset. According to the cut-off criteria |log2FC| ≥ 0.263 and (FDR) < 0.05, there were 11,530 genes identified as differentially expressed, of which 7794 were in the comparison CRC vs. Controls, 7434 were in the comparison Adenoma vs. Controls, and 7825 were in the comparison CRC vs. Adenoma.

Subsequently, wrapper methods were implemented for a more accurate selection. The first selection was done through the Boruta algorithm, which identified 240 important features. Then, Stepwise Regression was applied to the Boruta selected features. A total of 56 DEGs were selected as the optimal genes discriminating the three groups (Table S1).

### 3.2. Clustering Analysis

**Figure 2:**
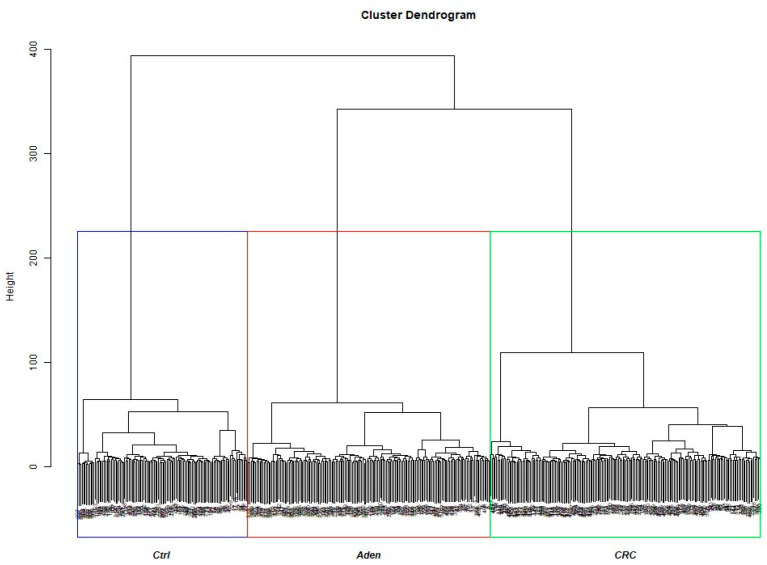
Ward hierarchical cluster dendrogram. The data were arranged in three cluster, confirming the hypothesis that gene expression could discriminate the 3 groups.

We firstly evaluated the optimal cluster number k through three different methods: Silhouette, Within Cluster Sum of Squares, Gap Statistic Method. For all the algorithms used, the optimal number of clusters was three (Figure S1). Then, for assessing the centroids of the three clusters, a Ward hierarchical cluster was developed. The cluster dendrogram generated shows that the three groups were clearly separate using the selected 56 DEGs (Figure 2).

**Figure 3:**
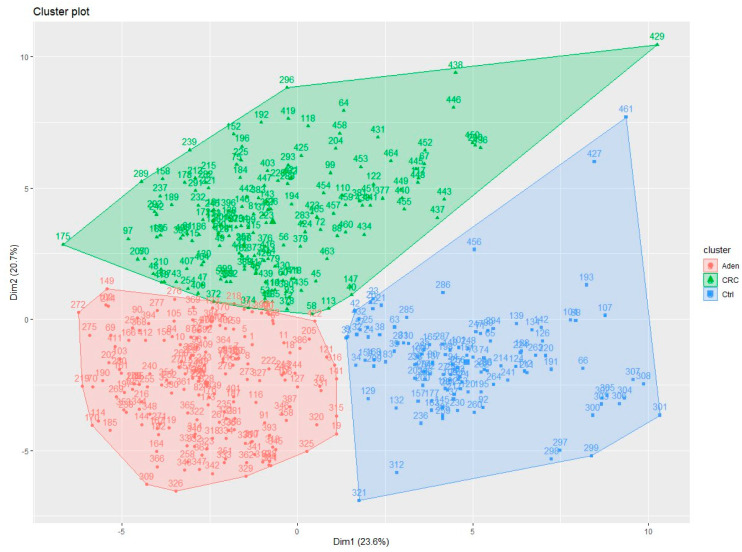
Cluster plot obtained through the K-Means algorithm.

This separateness was also confirmed by displaying the correlations among the selected 56 DEGs using the K-Means method (Figure 3).

Finally, we evaluated the correct assignment of the samples in each cohort, comparing the cluster label assigned by the K-Means algorithm to the true label of the sample. As shown in Table 1, most samples clustered in the correspondingly true group. Only a small part of the adenoma samples was clustered in the CRC group and vice versa, but this could be reasonable, since they represent the same disease but with different severity.

### 3.3. Machine Learning Analysis and Performance of the Gene-Based Classifier

**Figure 4:**
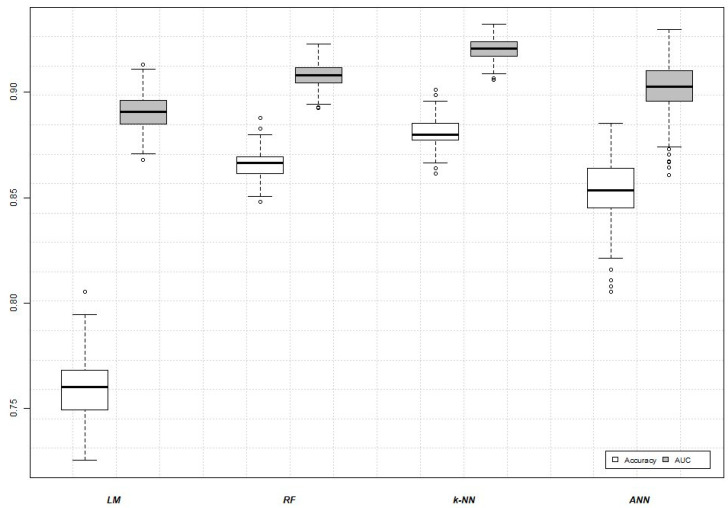
AUC and Accuracy for the four implemented models: LM, RF, k-NN, and ANN. Each boxplot was built through a 5-fold cross-validation procedure repeated 300 times. White dots represent the distribution outliers.

In order to build the four classifiers, we used the training set of samples composed of 375 observables and 56 selected features (genes). A five-fold cross-validation analysis repeated 300 times was computed. Then, we compared the performance of the LM, RF, k-NN, and ANN models based on Accuracy, AUC, Sensitivity, Precision, and *F*1 Score (Figure 4).

Classification performances of each model are summarized in Table 2.

**Figure 5:**
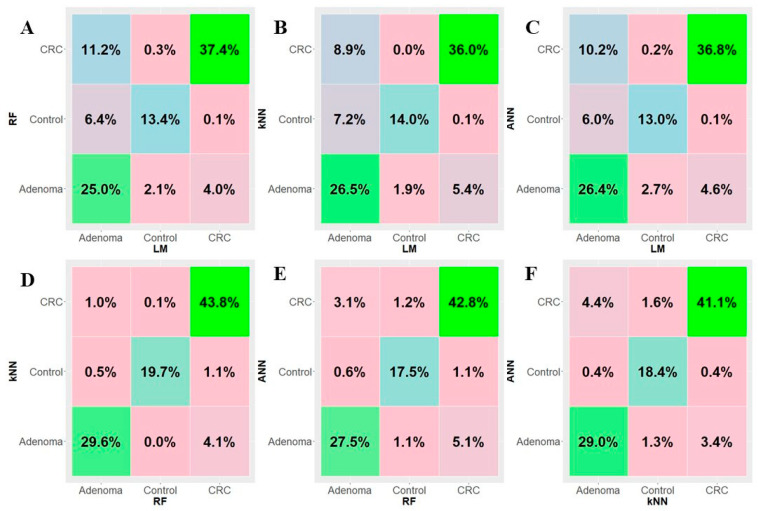
Contingency tables with pairwise comparisons of the implemented algorithms predictions averaged over 300 rounds of 5-fold cross-validation: RF vs. LM (panel (**A**)), k-NN vs. LM (panel (**B**)), ANN vs. LM (panel (**C**)), k-NN vs. RF (panel (**D**)), ANN vs. RF (panel (**E**)), ANN vs. k-NN (panel (**F**)).

The best classification method in terms of accuracy (88.06 ± 0.70) and AUC (92.04 ± 0.47) was k-NN. RF and ANN methods showed similar values, while the LM model was the least-performing method. In addition, we evaluated the consistency of the prediction of all four classifiers by means of pairwise contingency tables (Figure 5). Interestingly, in the panels k-NN vs. RF (Figure 5D), ANN vs. RF (Figure 5E), and ANN vs. k-NN (Figure 5F), the agreement between the classification models exceeded ≈90%, while, in the LM panels (Figure 5A–C), it exceeded ≈75%, which was still a high value.

Moreover, the Kruskall–Wallis test computed to study potential significant differences among the tested methodologies revealed no significant differences.

Finally, to confirm the relevance of the predictive models, we applied the four models on the validation cohorts composed of 90 samples, 30 randomly selected subjects for each group. As shown in Table 3, the k-NN model remained the best model in terms of performance, with 91.11% accuracy. Moreover, the sensitivity, precision, and *F*1 score values proved that the best performance was the k-NN model.

### 3.4. Pathway Analysis of the Gene-Based Classifier

Functional enrichment analysis on 56 DEGs with GSEA (FDR < 0.05) showed that DEGs were enriched in the following hallmarks: epithelial mesenchymal transition, hypoxia, angiogenesis, hedgehog signaling, IL2/STAT5 signaling, KRAS signaling (Table 4).

Moreover, Gene Ontology analysis for cellular component showed that the most significant were enriched in cell junctions and microvillus membranes (Table 5).

### 3.5. Selection of Biomarker Genes on Gene Expression and Survival Analysis

**Figure 6:**
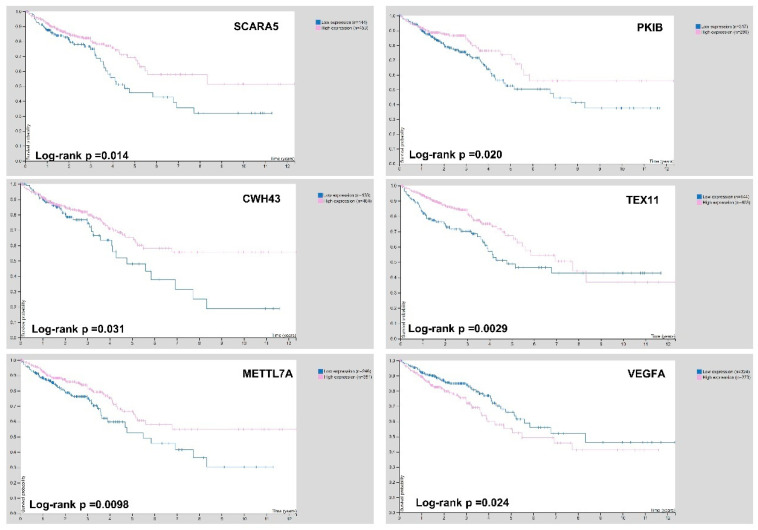
Kaplan–Meier survival analysis of SCARA5, PKIB, CWH43, TEX11, METTL7A, and VEGFA for CRC dataset downloaded from Pathology Atlas section of The Human Protein Atlas database.

In order to find the most significant biomarkers able to discriminate adenoma status from CRC, 56 DEGs were further filtered. The dataset was firstly normalized between 0 and 1; then, mean expression values in the three groups were evaluated for each gene. We identified the genes with a changed expression in a stepwise manner during the normal-adenoma–carcinoma sequence. Starting from 56 DEGs, we selected 17 genes (Table S2). Then, we compared the expression pattern of these genes in our analysis with RNA-seq data on TCGA. Their expression levels in the comparison CRC vs. normal mucosa evidenced the same trend. We could not verify the expression of these 17 genes in colorectal adenomas, since the TCGA database had no adenoma data. The shortlisted 17 genes were further filtered through the Pathology Atlas section of The Human Protein Atlas database, based on the overall survival analysis [36]. In total, six genes, namely SCARA5, PKIB, CWH43, TEX11, METTL7A, and VEGFA, were significantly correlated with the overall survival of CRC patients, suggesting that their expression is correlated with disease severity (Figure 6).

## 4. Discussion

CRC is characterized by variations in the molecular profile during the disease progression [37]. Previous studies have been conducted to identify genes involved in the progression of CRC [38]. Analysis of the transcriptome profiles in the colorectal normal mucosa–adenoma–carcinoma sequence may clarify the early mechanisms underlying CRC. To date, biomarkers routinely applied in clinical practice for discriminating adenoma from carcinoma are still lacking.

In recent years, the application of machine learning algorithms has provided new insight into early cancer detection [39,40,41]. In this study, using publicly available data from GEO, we applied an integrated machine learning and bioinformatics approach to identify new biomarker genes for the early diagnosis of CRC. Specifically, we analyzed gene expression data from 465 samples divided into three groups: 105 controls, 155 adenoma samples, and 205 CRC samples. Initially, a differential expression analysis was applied in order to find genes that are differentially expressed in adenoma and carcinoma conditions. Then, we performed a feature selection procedure to select relevant features for model construction. Starting from 11,530 DEGs, the feature selection procedure identified 56 DEGs as features serving for CRC classification.

Machine learning algorithms, namely Linear Model (LM), Random Forest (RF), k-Nearest Neighbors (k-NN), and Artificial Neural Network (ANN), were also used to classify the samples. The k-NN model was the best performing method in terms of accuracy and AUC. The other three provide a good performance, although RF and ANN provide a better performance compared to LM.

Similarly, the predictor-classifier demonstrated a high accuracy in the validation sets. In fact, the k-NN model still remained the best model in terms of performance, with an accuracy of 91.11%, and its accuracy was pretty balanced for the three classes. These results suggest that the classifier is robust. Thus, our study may establish a basis for further research into the early diagnosis of CRC.

Functional enrichment analysis on 56 DEGs revealed that they are involved in the epithelial mesenchymal transition, hypoxia, angiogenesis, hedgehog signaling, IL2/STAT5 signaling, KRAS signaling. Moreover, Gene Ontology analysis for cellular components showed that the most significant were enriched in cell junctions and microvillus membranes.

Among the 56 DEGs, we identified 17 genes with an ascending or descending trend through the normal mucosa–adenoma–carcinoma sequence. In addition, using the survival information of the TCGA database, we selected six DEGs related to patient prognosis. A functional review of the selected genes (SCARA5, PKIB, CWH43, TEX11, METTL7A, and VEGFA) demonstrated that all of them are reported to be related to the pathogenesis of CRC. In scavenger receptor class A, member 5 (SCARA5) is a tumor suppressor gene that was downregulated in many cancer types, including CRC [42,43,44]. Protein kinase (cAMP-dependent, catalytic) inhibitor beta (PKIB) promotes cell proliferation [45] and has been shown to be upregulated in lung cancer. To the best of our knowledge, there are no studies correlating the expression levels of PKIB with CRC. In our analysis, its expression pattern decreased from normal mucosa to adenoma and carcinoma. Our findings are consistent with TCGA RNA-seq data. Cell Wall Biogenesis 43 C-Terminal Homolog (CWH43) has been reported to be downregulated in colorectal tumor tissues, although little is known about its function [46]. Testis-expressed gene 11 (TEX11) is a germ cell-specific gene [47], and the formation of crossovers and mutations in the TEX11 gene may be a genetic cause of infertility in men [48]. In CRC, in accordance with our analysis, TEX11 was downregulated in patients compared with healthy controls [49]. Luo et al. hypothesized that, since TEX11 is an X-linked gene, its differential expression may be a genetic cause that could explain the higher incidence of CRC in males. Methyltransferase-like protein 7A (METTL7A) belongs to the human methyltransferase-like protein family, and the low METTL7A expression has been associated to cancer aggressiveness and progression in various tumors, including CRC [50,51,52,53]. Vascular endothelial growth factor A (VEGFA) and its receptors have been identified as major mediators of angiogenesis, which is crucial for tumor invasiveness [54]. VEGFA was upregulated in some solid tumors, including primary and metastatic colorectal carcinoma [55,56]. Therefore, in CRC, the levels of VEGFA are associated with poor prognosis [57], and the use of bevacizumab (a specific anti-VEGF drug) has led to increased survival times [58].

This study indicates novel potential targets for the early diagnosis of CRC. Nevertheless, it has several limitations. Firstly, the study was based on a small sample size; future studies are needed to validate and improve the predictor-classifier in a larger multicenter prospective patient cohort. Secondly, data in the GEO database were derived from different experimental platforms, and although internal standardization has been performed, the results could be heterogeneous. Thirdly, our results were based on tissue samples, which still remains an invasive procedure. Future investigations are needed to demonstrate whether the identified genes could be detected in blood to allow a non-invasive diagnosis and prognosis of the disease.

In conclusion, we identified six DEGs involved in the normal colorectal mucosa–adenoma–carcinoma sequence associated with CRC patients’ prognosis. Our results demonstrate the robust diagnostic performance of the gene-based classifier in the training and validation cohorts, confirming its potential clinical value. These findings may help to elucidate the molecular mechanisms involved in the onset and development of CRC, providing the basis for the identification of potential biomarkers for early diagnosis and of new therapeutic targets.

## Figures and Tables

**Table 1:** Contingency table between the true label and the cluster one.

		Cluster Label		
		Adenoma	CRC	Control
**True Label**	**Adenoma**	139	10	6
	**CRC**	36	155	14
	**Control**	1	4	100

**Table 2:** Train classification performances with respective standard deviations: Accuracy, AUC, Sensitivity, Precision, and *F*1 score for the four models LM, RF, k-NN, and ANN for each class.

Accuracy and AUC (%)				
	LM	RF	k-NN	ANN
Accuracy	75.85±1.35	86.59±0.68	88.06±0.70	85.37±1.40
AUC	89.05±0.82	90.81±0.53	92.04±0.47	90.25±1.23
**Sensitivity (%)**				
**Class**	**LM**	**RF**	**k-NN**	**ANN**
Control	67.72±3.41	90.52±1.49	97.19±0.45	86.11±3.57
Adenoma	78.15±2.24	80.59±1.39	85.26±1.19	82.52±2.52
CRC	77.69±1.75	89.19±0.88	86.15±1.15	87.08±1.84
**Precision (%)**				
**Class**	**LM**	**RF**	**k-NN**	**ANN**
Control	85.28±2.70	90.98±0.96	91.32±1.03	89.82±2.50
Adenoma	61.12±1.72	86.40±1.25	84.19±1.41	81.61±2.37
CRC	87.43±1.38	84.95±0.92	89.45±0.83	86.34±1.76
***F*** **1 Score (%)**				
**Class**	**LM**	**RF**	**k-NN**	**ANN**
Control	75.44±2.64	90.74±0.97	94.16±0.60	87.87±2.32
Adenoma	68.58±1.65	83.39±1.00	84.72±1.02	82.03±1.86
CRC	82.26±1.24	87.02±0.70	87.76±0.78	86.69±1.33

**Table 3:** Test classification performances: Accuracy, Sensitivity, Precision and *F*1 score for the four models LM, RF, k-NN, and ANN for each class.

Accuracy (%)				
	LM	RF	k-NN	ANN
Accuracy	67.78	89.26	91.11	86.71
**Sensitivity (%)**				
**Class**	**LM**	**RF**	**k-NN**	**ANN**
Control	63.33	82.78	93.33	85.39
Adenoma	73.33	93.57	90.00	87.12
CRC	66.67	91.43	90.00	87.61
**Precision (%)**				
**Class**	**LM**	**RF**	**k-NN**	**ANN**
Control	90.48	87.36	87.50	88.15
Adenoma	51.16	96.46	93.10	87.08
CRC	76.92	84.60	93.10	85.61
***F*1 Score (%)**				
**Class**	**LM**	**RF**	**k-NN**	**ANN**
Control	74.51	84.96	90.32	86.62
Adenoma	60.27	94.98	91.53	86.99
CRC	71.43	87.85	91.53	86.47

**Table 4:** Gene Set Enrichment analysis using the hallmark gene set.

Gene Set Name	*p*-Value	FDR *q*-Value
HALLMARK_EPITHELIAL_MESENCHYMAL_TRANSITION	3.65 × 10^−7^	1.83 × 10^−5^
HALLMARK_HYPOXIA	1.76 × 10^−4^	4.41 × 10^−3^
HALLMARK_ANGIOGENESIS	1.16 × 10^−3^	1.45 × 10^−2^
HALLMARK_HEDGEHOG_SIGNALING	1.16 × 10^−3^	1.45 × 10^−2^
HALLMARK_IL2_STAT5_SIGNALING	2.71 × 10^−3^	1.96 × 10^−2^
HALLMARK_KRAS_SIGNALING_UP	2.75 × 10^−3^	1.96 × 10^−2^
HALLMARK_XENOBIOTIC_METABOLISM	2.75 × 10^−3^	1.96 × 10^−2^

**Table 5:** Gene Ontology analysis on 56 DEGs identified as a classifier of normal–adenoma–carcinoma status.

Term Name	Term ID	T	U	*p*_adj
cell junction	GO:0030054	2107	18964	6.228 × 10^−4^
microvillus membrane	GO:0031528	27	18964	7.055 × 10^−3^
cell projection membrane	GO:0031253	346	18964	3.373 × 10^−2^

## Data Availability

Not applicable.

## Supplementary Materials

The following are available online at www.mdpi.com/article/10.3390/biomedicines9121937/s1, Figure S1: Average Silhouette, Total within Sum of Squares and Gap Statistic in function of the number of clusters. For all three methods, three is the optimal number of clusters. Table S1: List of 56 selected features (genes) with corresponding logFC (Fold-change) and adjusted-*p* value for the three comparisons. Table S2. List of 17 selected genes with their expression levels in the three groups.
