# Supplementary figures and images for: A Gene-Based Machine Learning Classifier Associated to the Colorectal Adenoma—Carcinoma Sequence
Source: Biomedicines. 2021 Dec 17;9(12):1937. doi: 10.3390/biomedicines9121937 (PMC8698794; doi:10.3390/biomedicines9121937)

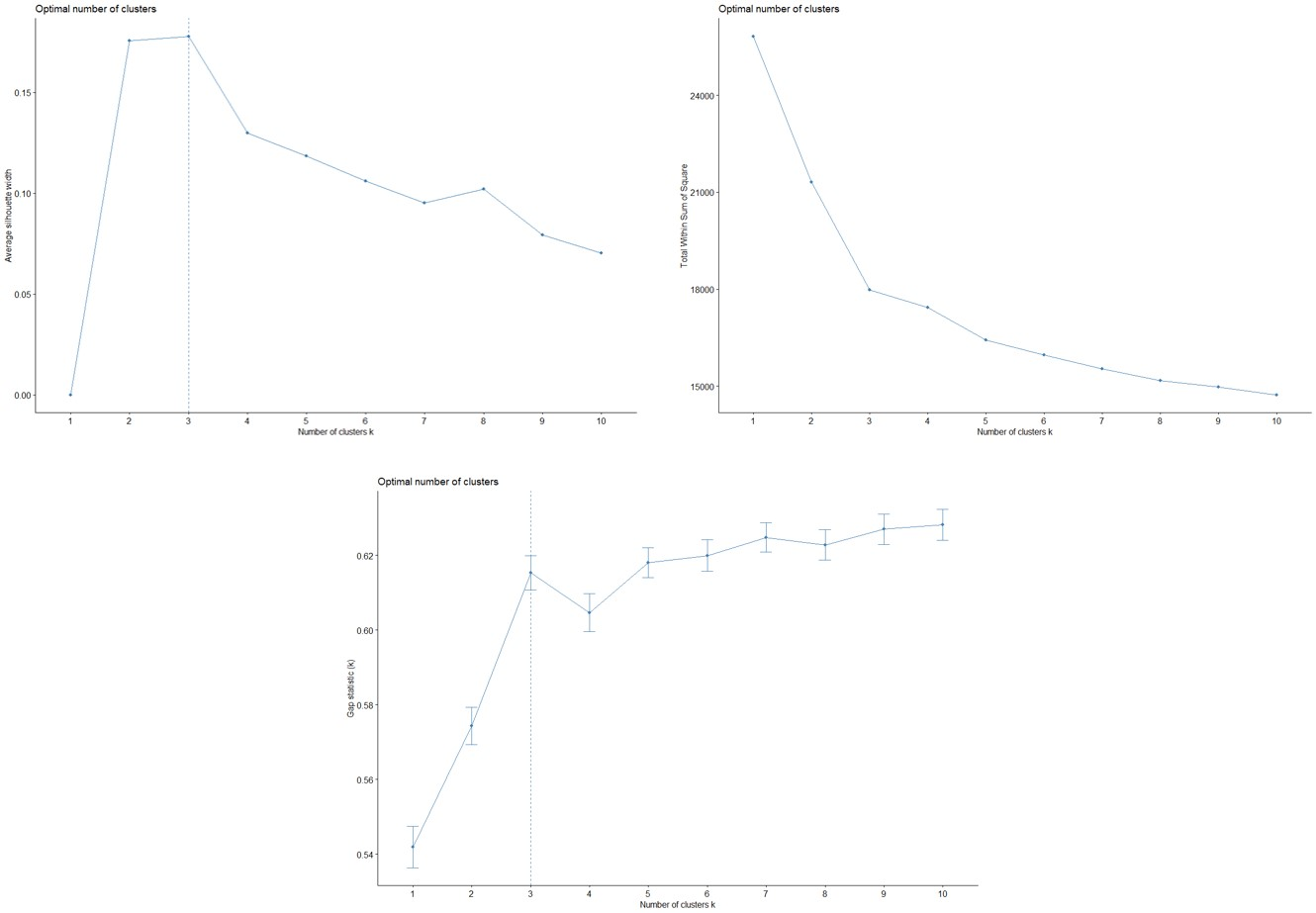

Supplement: Supplementary file 1 [file biomedicines-09-01937-s001.zip › Supplementary Files/Figure S1.tiff]
